# Pharmacokinetic–pharmacodynamic evaluation of therapeutic efficacy of intramuscular amoxicillin–florfenicol in olive flounder

**DOI:** 10.4142/jvs.25365

**Published:** 2026-07-06

**Authors:** Eun-Hee Kang, Hae-Yeon Cho, Ling Gui, Ga-Yeong Lee, Eon-Bee Lee, Seung-Chun Park

**Affiliations:** 1 Institute for Veterinary Biomedical Science, College of Veterinary Medicine, Kyungpook National University, Daegu 41566, Korea.; 2 Department of Aquatic Life Medicine, Pukyong National University, Busan 48513, Korea.

**Keywords:** Drug synergism, *Edwardsiella tarda* infections, *Paralichthys olivaceus*, pharmacokinetics, PK-PD index

## Abstract

**Importance:**

*Edwardsiella tarda* induces severe systemic infections in olive flounder (*Paralichthys olivaceus*), requiring long-acting parenteral therapies to ensure optimal antimicrobial exposure. While amoxicillin trihydrate (AMXT) and florfenicol (FFC) are effective monotherapies, the pharmacokinetic-pharmacodynamic (PK-PD) rationale for their combined intramuscular administration remains insufficiently characterized.

**Objective:**

To evaluate the PK-PD profile, synergistic activity, and therapeutic efficacy of a fixed-dose intramuscular AMXT–FFC combination against *E. tarda* in olive flounder.

**Methods:**

Minimum inhibitory concentration (MIC) and time–kill assays were conducted. Olive flounder were administered amoxicillin (AMX, 10 mg/kg), FFC (10 mg/kg), or their combination (10 + 10 mg/kg) via intramuscular injection. PK parameters were estimated using noncompartmental analysis. PK-PD indices, including %time above MIC (%T > MIC) and area under the concentration–time curve (AUC)/MIC, were calculated based on the combined MIC (0.5 µg/mL). Therapeutic efficacy was assessed using an dose causing 70% mortality challenge model (3.7 × 10^6^ colony-forming units [CFU]/fish).

**Results:**

The AMXT–FFC combination exhibited synergism (ΣFIC = 0.625), achieving a ≥ 3 log_10_ CFU/mL reduction within 24 h. Co-administration significantly elevated C_max_ and prolonged t_1/2_, reaching 34.12 µg/mL and 33.27 h for AMX, and 31.59 µg/mL and 13.94 h for FFC, respectively. PK-PD integration showed that PK-PD indices met prespecified efficacy targets (AMX: 100% T > MIC; FFC: AUC/MIC = 1,610.82). *In vivo*, the combination significantly improved survival compared to controls (75% vs. 27.5%; relative percent survival = 65.5%, *p* < 0.05).

**Conclusions and Relevance:**

A single intramuscular administration of the AMXT–FFC combination provides synergistic antibacterial activity, prolongs exposure, and achieves prespecified PK-PD targets under the controlled experimental conditions.

## INTRODUCTION

Aquaculture is crucial in global food security, providing a sustainable and increasingly important source of high-quality animal protein. Among marine species cultivated in aquaculture, the olive flounder (*Paralichthys olivaceus*) represents a major commodity in East Asia—particularly in the Republic of Korea, where it accounts for a significant portion of total fisheries production—and in Japan and China. This importance is attributed to its rapid growth rate, efficient feed conversion, and strong market demand [1,2]. However, the intensification of aquaculture leads to a rise in bacterial diseases—particularly those caused by *Edwardsiella tarda*, *Vibrio* spp., and *Streptococcus iniae*—resulting in substantial economic losses in flounder farming [3,4,5]. This growing disease burden, often characterized by systemic infections and high mortality rates, underscores the need for rapid and accurate diagnostic tools to facilitate timely clinical intervention [6].

Antibiotics remain the primary treatment for bacterial infections in aquaculture, with both monotherapy and combination regimens widely used in commercial finfish production [6,7,8,9,10,11]. Nonetheless, inappropriate or excessive antibiotic use reduces long-term treatment efficacy, promotes resistant bacterial strain emergence, and poses ecological and public health concerns [8,12,13]. These challenges highlight the need for evidence-based strategies for preserving the effectiveness of existing antimicrobials and support the development of novel agents tailored to evolving resistance patterns in aquaculture systems [9,14,15,16].

Edwardsiellosis, caused by the Gram-negative bacterium *E. tarda*, is a severe systemic disease characterized by septicemia, hemorrhage, and exophthalmia, affecting economically important species, including turbot, tilapia, and olive flounder [5,17,18,19]. The pathogenicity of *E. tarda* is multifactorial, involving immune evasion, intracellular persistence, and metabolic adaptability, which hinder bacterial clearance and complicate therapeutic management [14,15,18]. Effective disease control requires precise antimicrobial selection and dosing guided by pharmacokinetic (PK) and pharmacodynamic (PD) principles. Among drug-delivery routes in fish, intramuscular injection provides accurate dosing, reduces variability in drug intake and absorption, and minimizes environmental contamination compared with medicated feed. Amoxicillin trihydrate (AMXT), a β-lactam antibiotic approved for human and veterinary use, exhibits broad-spectrum antibacterial activity [20]. Studies report the PK-PD properties and withdrawal periods of amoxicillin (AMX) sodium in olive flounder [21], while additional research has elucidated AMX kinetics in tilapia [22,23] and cultured flounder [24]. AMXT, a relatively low-solubility formulation, exhibits a prolonged apparent half-life and sustained plasma concentrations in fish species, potentially reducing dosing frequency. Florfenicol (FFC), a fluorinated derivative of thiamphenicol, is another widely used antimicrobial agent in aquaculture [25,26,27,28]. While AMXT and FFC are individually effective, the PK, PD, and therapeutic profiles of their approved injectable fixed-dose combination in olive flounder remain poorly characterized. Therefore, this study aims to (i) evaluate the *in vitro* synergy of AMXT and FFC against *E. tarda*, (ii) compare PK profiles after single-agent versus combined intramuscular administration, and (iii) assess the therapeutic efficacy of the AMXT–FFC combination in olive flounder experimentally infected with *E. tarda*. These findings may provide mechanistic insights and practical evidence to support the rational use of the AMXT–FFC combination in aquaculture, thereby contributing to improved disease management and food safety.

## METHODS

### Chemicals and reagents

AMXT (≥ 87% purity, poorly water-soluble, equivalent to 10 mg/kg AMX base) and FFC (≥ 99% purity, 10 mg/kg active base) were supplied by Daesung Microbiological Institute (Korea). Dosages were calculated based on the active base equivalent, with adjustment for the purity of each raw material. Analytical standards of AMXT, FFC, and FFC amine were obtained from Sigma-Aldrich (USA). High-performance liquid chromatography-grade acetonitrile, methanol, and ethyl acetate were acquired from J.T. Baker (USA). Disodium phosphate, trichloroacetic acid, hexane, and glacial acetic acid were of analytical grade. All aqueous solutions were prepared using ultrapure water (Milli-Q; Merck Millipore, USA). Stock solutions (200 mg/mL) were prepared using sterile distilled water for AMXT and dimethylformamide for FFC, then diluted with the vehicle immediately before administration.

### Bacterial strain and culture conditions

*E. tarda* FP2018 (KCTC 11145BP) was obtained from the Korean Collection for Type Cultures (Korea). Species identity was confirmed via multiplex polymerase chain reaction (PCR) employing species-specific primers targeting *E. tarda*, *S. iniae*, and *Streptococcus parauberis*. Bacterial cultures were incubated in tryptic soy broth (Difco, USA) at 28°C with agitation at 180 rpm for 18–20 h, until an optical density at 600 nm of approximately 0.5 (~1 × 10^8^ colony-forming units [CFU]/mL) was reached. Culture purity was verified through Gram staining and examining colony morphology on tryptic soy agar (TSA). Fresh working cultures were prepared from −70°C Microbank stocks (Pro-Lab Diagnostics, Canada) before each experiment.

### *In vitro* susceptibility and time–kill assay

Minimum inhibitory concentrations (MICs) of AMXT, FFC, and their combination against *E. tarda* FP2018 were determined via broth microdilution following Clinical and Laboratory Standards Institute VET03-A guidelines (2020) [29]. The MIC values for AMXT, FFC, and their combination were 4 µg/mL, 1 µg/mL, and 0.5 µg/mL, respectively. Time–kill assays were conducted at 1×, 2×, and 4× MIC in Mueller–Hinton broth at 28°C for 24 h. Samples were collected at predetermined intervals for viable bacterial counts. Bactericidal activity was defined as a ≥ 3 log_10_ CFU/mL reduction from baseline, whereas synergy was defined as a ≥ 2 log_10_ CFU/mL reduction relative to the most active monotherapy.

### Animal care and husbandry

All experimental procedures were approved by the Institutional Animal Care and Use Committee of Kyungpook National University (approval No. KNU-2015-0020) and complied with national animal welfare regulations. Overall, 440 clinically healthy olive flounder (*P. olivaceus*; 120 ± 12 g, 25–30 cm in length) were maintained at Dongwon Fisheries (Korea) and used for the PK and therapeutic efficacy studies. For the PK study, olive flounders (n = 150) were equally distributed into three 400 L tanks (n = 50 per tank) and assigned to the following treatment groups: AMXT (10 mg/kg AMX base), FFC (10 mg/kg FFC base), and an AMXT–FFC combination (10 + 10 mg/kg). For the efficacy study (n = 290), an initial challenge test was conducted with 50 fish, divided into five glass tanks (n = 10 per tank), to determine the dose causing 70% mortality (LD_70_) of *E. tarda*. Subsequently, the remaining 240 fish were challenged with the established LD_70_ dose to evaluate therapeutic efficacy. These fish were stocked into six 400 L tanks at a density of 40 fish per tank and divided into the following groups: (1) uninfected control, (2) vehicle control, (3) AMXT-treated, (4) FFC-treated, (5) AMXT–FFC-treated, and (6) infected control. All experimental groups were maintained in separate tanks under uniform environmental conditions, including water temperature, pH, and dissolved oxygen levels. All fish were acclimated for at least 14 days in flow-through seawater systems and fed a commercial diet once daily, with feeding withheld 24 h before dosing. Water quality parameters were maintained as follows: temperature, 23 ± 2°C; pH, 8.11 ± 0.23; dissolved oxygen, 8.69 ± 0.21 mg/L; total ammonia nitrogen, 0.035 ± 0.010 mg/L; and nitrite nitrogen, 0.028 ± 0.008 mg/L (Supplementary Table 1). No mortality occurred during acclimation.

### PK Study

#### Drug administration and blood collection

Fish were administered intramuscular injections of AMXT (10 mg/kg AMX base), FFC (10 mg/kg), or the AMXT–FFC combination (10 + 10 mg/kg) into the dorsal musculature. At each post-administration sampling time (0, 0.5, 1, 3, 6, 9, 12, 18, 24, and 48 h), five fish were euthanized, and ~0.5 mL of blood was collected from the caudal vein. The sample sizes were selected to ensure statistical validity and regulatory compliance, with n = 5 or 10 per time point for PK sampling, adhering to VICH GL52 and European Medicines Agency guidelines for terminal sampling in small teleosts—a design supported by established aquatic PK methodology [30]. Blood samples were centrifuged at 12,000 × g for 10 min at 4°C, and plasma was stored at −80°C until analysis. Anesthesia was omitted to prevent interference with PK parameters.

#### Plasma sample preparation and high-performance liquid chromatography analysis

Plasma samples were divided into two aliquots for separate analysis using a Hewlett-Packard 1100 HPLC system (Agilent, USA). The system utilized an Eclipse Plus C18 column (150 × 4.6 mm, 3.5 µm) eluted with 50 mM phosphate buffer (pH 3.0) and acetonitrile (95:5, v/v) at 229 nm for AMX, and an HP ODS column (200 × 4.6 mm, 5 µm) eluted with acetonitrile and water (25:75, v/v) at 224 nm for FFC. The flow rate for both analyses was maintained at 1.0 mL/min. For AMX extraction, 0.5 mL of plasma was mixed with 2.5 mL of acetonitrile, vortexed for 10 min, and centrifuged at 12,000 × g for 10 min. The supernatant was freeze-dried under nitrogen and reconstituted in 0.1 mL of the mobile phase before injection. Supplementary Fig. 1 provides the analytical validation results. Calibration curves showed linearity over the concentration ranges of 0.05–10 µg/mL for AMX and 0.1–50 µg/mL for FFC (r^2^ > 0.99). The limits of detection and quantification (LOQ) were 0.05 0.1 µg/mL and 0.1 µg/mL for AMX, respectivey, whereas the LOQ for FFC was 0.1.

### PK analysis

Noncompartmental PK analysis was performed using mean concentration–time data with Phoenix WinNonlin 8.3 software (Certara, USA). The calculated PK parameters included maximum plasma concentration (C_max_), time to reach C_max_ (T_max_), area under the concentration–time curve from zero to infinity (AUC_0–∞_), elimination half-life (t_½_), mean residence time (MRT_0–∞_), apparent clearance (Cl/F), and apparent volume of distribution (Vz/F). These PK parameters provide descriptive estimates appropriate for composite sampling in aquatic species [30].

### Dose justification and PK/PD analysis

Dose selection was based on PK-PD principles connecting systemic drug exposure to bacterial susceptibility. For β-lactam antibiotics (AMXT), the target PD index was the percentage of time that plasma concentrations exceeded the MIC (%T > MIC) of at least 40%–50% over 24 h and ≥60%–70% for severe infections. For phenicols (FFC), the primary PK-PD target included an AUC/MIC ratio ≥ 125 and a C_max_/MIC ratio of ≥ 8–10. These indices were calculated using experimentally derived PK parameters and a combined MIC value of 0.5 µg/mL.

### *In vivo* therapeutic efficacy

#### Median lethal dose (LD_50_) and LD_70_ determination

To determine the LD_70_ of *E. tarda*, five groups of 10 fish each were intraperitoneally injected with 0.1 mL of the pathogen at concentrations of 0 (control), 10^3^, 10^5^, 10^7^, and 10^9^ CFU/fish, and mortality was monitored for 14 days. The LD_50_ and LD_70_ values were estimated using nonlinear sigmoidal regression analysis in GraphPad Prism 10 software (GraphPad Software, USA). The LD_70_ value (3.7 × 10^6^ CFU/fish) was selected for the subsequent therapeutic efficacy trial.

#### Therapeutic efficacy trial

The therapeutic efficacy trial was conducted via intraperitoneal injection of *E. tarda* at a dose of 3.7 × 10^6^ CFU/fish, corresponding to the established LD_70_. The bacterial concentration was verified using serial dilution and plate counting on TSA, and the inoculum was adjusted to a final concentration of 3.7 × 10^6^ CFU/fish for intraperitoneal administration. To evaluate the therapeutic effect, antibiotic treatments were administered intramuscularly 48 h post-infection. Fish survival was monitored for 14 days post-infection, and the relative percent survival (RPS) was calculated to evaluate treatment efficacy. Mortality was recorded based on predefined criteria, including loss of equilibrium, unresponsiveness to external stimuli, and cessation of opercular movement. At 3 days, 7 days, and 14 days post-infection, five fish per group were euthanized with MS-222 (200 mg/L). The liver, kidney, spleen, brain, and eye tissues were collected, homogenized in phosphate-buffered saline, plated on TSA, and incubated at 28°C for 24–48 h. Colonies were characterized based on morphology, Gram staining, and confirmation by *gyr*B PCR amplification (415 bp). Multiplex PCR was performed to exclude the presence of *S. iniae* and *S. parauberis*. Gross pathological lesions, including hemorrhage, congestion, and necrosis, were scored using a 0–3 scale. Isolates were preserved at −70°C in Microbank vials for subsequent confirmation. Treatment allocation was randomized, and investigators responsible for lesion scoring and bacteriological confirmation were blinded to group identity during outcome assessment.

### Statistical analyses

Statistical analyses were conducted using GraphPad Prism 10 (GraphPad Software) and R version 4.4.1 (R Foundation for Statistical Computing, USA). Survival outcomes were analyzed using Kaplan–Meier curves and compared using the log-rank tests. Normality was assessed using the Shapiro–Wilk test, while homogeneity of variance was evaluated using Levene’s test. Normally distributed data were analyzed using one-way analysis of variance with Tukey’s *post hoc* test, whereas non-normal data were analyzed using Kruskal–Wallis testing with Dunn’s *post hoc* test and Holm correction for multiple comparisons. Multiplicity across organs and time points was controlled using the Holm procedure. PK parameters were estimated from mean concentration–time data under a composite sampling design and are presented descriptively. However, AUC_last_ values for single and combination treatments were compared using the Bailer–Satterthwaite method, appropriate for sparse composite data. Other PK parameters were not formally compared statistically. All statistical tests were two-sided, and *p* values < 0.05 were considered statistically significant for inferential analyses.

## RESULTS

### *In vitro* susceptibility and time–kill assay

**Fig. 1:**
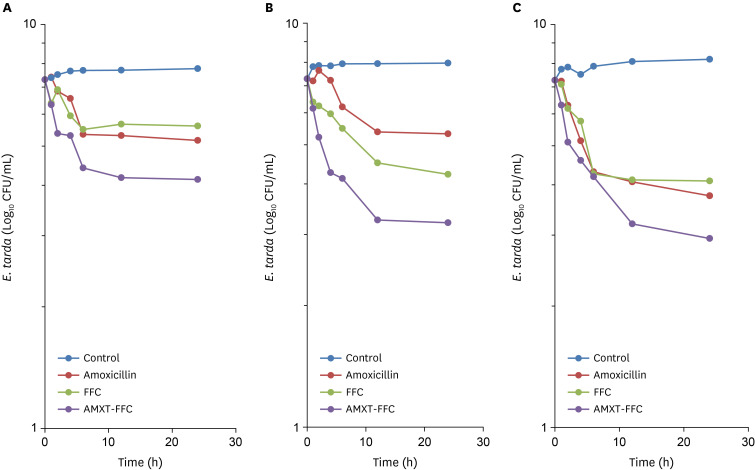
Time–kill kinetics of AMXT, FFC, and their fixed 1:1 combination (AMXT–FFC) against *Edwardsiella tarda* FP2018 at 1×, 2×, and 4× MIC. Each data point represents the mean log_10_ CFU/mL ± SD (n = 3). The combination exhibited rapid and sustained bactericidal activity, achieving a ≥ 3 log_10_ CFU/mL reduction within 24 h and an additional ≥ 2 log_10_ CFU/mL compared to the most active single agent, indicating synergy (ΣFIC = 0.625). AMXT, amoxicillin trihydrate; FFC, florfenicol; MIC, minimum inhibitory concentration; CFU, colony-forming units; SD, standard deviation; ΣFIC, sum of fractional inhibitory concentration index.

The MICs of AMXT, FFC, and their fixed 1:1 combination against *E. tarda* FP2018 were 4 µg/mL, 1 µg/mL, and 0.5 µg/mL, respectively (Supplementary Table 2). Time–kill assays demonstrated that bactericidal activity was both concentration- and time-dependent. At 1× MIC, AMXT alone reduced viable bacterial counts to 1.46 × 10^5^ CFU/mL after 24 h, while FFC reduced counts to 4.03 × 10^5^ CFU/mL. Conversely, the AMXT–FFC combination at 1× and 2× MIC produced rapid, sustained bactericidal activity against *E. tarda*. Specifically, the combination achieved near-complete bacterial eradication (~1.36 × 10^4^ CFU/mL) within 12–24 h, meeting the bactericidal threshold of a ≥ 3 log_10_ CFU/mL reduction relative to the initial inoculum (Fig. 1). At 2× MIC, viable counts decreased to ~1.33 × 10^4^ CFU/mL within 6 h and remained suppressed through 24 h, indicating pronounced time-dependent killing. At 4× MIC, the 24-h viable count further declined to 8.77 × 10^2^ CFU/mL, indicating concentration-dependent bacterial clearance. Across all tested concentrations, the combination consistently achieved ≥ 2 log_10_ greater reduction than the most active single agent, confirming pharmacodynamically significant synergy. A fractional inhibitory concentration index of 0.625 further supported this synergistic effect (Fig. 1).

### Chromatographic specificity and method validation

The specificity of the high performance liquid chromatography method was evaluated by comparing the chromatograms of blank plasma, standard-spiked plasma, and experimental plasma samples. Method linearity was validated over concentration ranges of 0.05–10 µg/mL for AMX and 0.1–50 µg/mL for FFC. Supplementary Fig. 1A shows the calibration curves for both antibiotics, exhibiting excellent linearity, with coefficients of determination (R^2^) > 0.9998. Additionally, the inter-assay precision was evaluated, with the coefficient of variation values ranging from 0.226% to 2.844% for AMX and 0.864% to 5.170% for FFC across the tested concentrations.

Supplementary Fig. 1B and C show that AMX and FFC are well resolved with sharp, symmetrical peaks at their respective detection wavelengths (229 nm and 224 nm). No significant interfering peaks from endogenous plasma components were observed at the analyte retention times, confirming the selectivity of the method for plasma analysis. These findings indicate that the developed method provides consistent quantitative performance with high precision and linearity.

### PK profiles

**Fig. 2:**
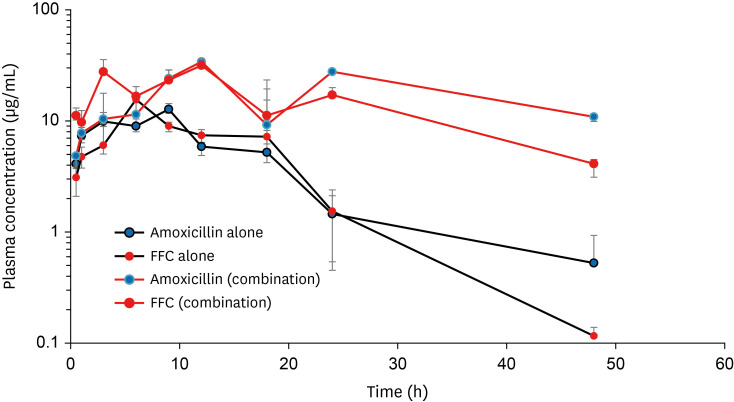
Plasma concentration–time profiles of AMXT and FFC in olive flounder following a single intramuscular dosing (10 mg/kg each), administered alone or in combination (AMXT–FFC). Data are presented as mean ± SD (n = 5 fish per time point). AMXT, amoxicillin trihydrate; FFC, florfenicol; SD, standard deviation.

The AMXT formulation exhibited slower absorption (T_max_ = 2.44 h) and a longer apparent terminal half-life (17.2 h) than more water-soluble β-lactam salts. Systemic exposure parameters showed that coadministration significantly increased AMX AUC_last_ under the present experimental conditions (Supplementary Table 3). Despite limitations imposed by the composite sampling design, the propagated standard error–based analysis (Bailer–Satterthwaite approach) supports increased systemic exposure. This profile may reflect formulation-dependent absorption characteristics; however, the composite sampling design prevents full separation of absorption and elimination processes. AMXT and FFC maintained plasma concentrations above their respective MICs for ≥ 48 h. Following intramuscular injection, the mean plasma concentration–time profiles of AMXT and FFC showed rapid absorption followed by a gradual decline, consistent with biphasic disposition typical of extravascular administration (Fig. 2). In the combination group, absorption was slightly delayed, and systemic exposure was moderately higher than that of the single-agent administration, suggesting slower release and prolonged persistence at the injection site. PK was evaluated using a noncompartmental analysis (NCA) model owing to composite sampling and study design limitations for estimating interindividual variability or compartmental distribution parameters. NCA provides sufficient, model-independent estimates of exposure (AUC, MRT) and disposition parameters (t_1/2_, Cl/F, and Vz/F). This approach is widely used to characterize antibiotic PK in fish species, particularly when the data adequately capture absorption and elimination phases.

Table 1 summarizes PK parameters following single administration of AMXT (10 mg/kg) and FFC (10 mg/kg). For AMXT, the mean C_max_ was 12.73 µg/mL at 9.0 h (T_max_) with an elimination t_1/2_ of 9.0 h, while FFC reached a C_max_ of 15.60 µg/mL at 6.0 h (T_max_) with a t_1/2_ of 6.0 h. Compared to single administration, coadministration significantly altered the disposition of both drugs, resulting in increased systemic exposure.

**Table 1:** Mean pharmacokinetic parameters of AMXT and FFC following a single intramuscular dose (10 mg/kg each), administered alone or in combination

Parameters	AMXT alone	FFC alone	AMXT combination	FFC combination
λz (1/h)	0.08	0.12	0.02	0.05
t_1/2_ (h)	9.00	6.00	33.27	13.94
T_max_ (h)	9.00	6.00	12.00	12.00
C_max_ (μg/mL)	12.73	15.60	34.12	31.59
C_last_ (μg/mL)	0.53	0.12	10.89	4.12
AUC_last_ (μg·h/mL)	186.95	197.56	908.16	722.55
AUC_0−∞_ (μg·h/mL)	193.83	198.60	1,430.89	805.41
AUC%_Extrap_ (%)	3.55	0.52	36.53	10.29
AUMC_0−∞ _(μg·h^2^/mL)	2,717.05	2,437.33	70,241.12	18,245.34
MRT_0−∞_	14.02	12.27	49.09	22.65
Vz/F (mL/kg)	669.98	436.18	335.46	249.71
Cl/F (mL/h/kg)	51.59	50.35	6.99	12.42

AMXT, amoxicillin trihydrate; FFC, florfenicol. *λz*, terminal elimination rate constant; t_1/2_, half-life; T_max_, time to maximum plasma concentration; C_max_, maximum plasma concentration; C_last_, last measurable concentration; AUC_last_, area under the concentration–time curve from time 0 to last measurable time point; AUC_0–∞_, area under the concentration–time curve from time 0 extrapolated to infinity; AUC_%Extrap_, percentage of AUC extrapolated beyond last measurable concentration; AUMC_0–∞_, area under the first moment curve from time 0 to infinity; MRT_0–∞_, mean residence time from time 0 to infinity; Vz/F, apparent volume of distribution normalized by bioavailability; Cl/F, apparent clearance normalized by bioavailability.

Coadministration significantly increased AUC_last_ for AMX and FFC (Supplementary Table 3). AMX AUC_last_ increased from 186.95 ± 22.73 µg/mL to 908.16 ± 32.75 µg·h/mL (Z = −18.091, *p* < 0.001), and FFC AUC_last_ increased from 197.56 ± 32.75 µg/mL to 722.55 ± 60.07 µg·h/mL (Z = −7.673, *p* < 0.001). Consistent with increased exposure, plasma concentrations of both agents remained above the synergistic MIC (0.5 µg/mL) for > 48 h postinjection.

Specifically, in the combination group, AMX reached a C_max_ of 34.12 µg/mL (2.68-fold increase) with T_max_ extended to 12.0 h, while elimination t_1/2_ and AUC_0−∞_ were substantially prolonged to 33.27 h and 1,430.89 µg·h/mL, respectively (Table 1). Similarly, FFC in combination reached a C_max_ of 31.59 µg/mL (2.03-fold increase) at 12.0 h, with elimination t_1/2_ and AUC_0−∞_ increased to 13.94 h and 805.41 µg·h/mL. These changes were accompanied by significantly increased MRT_0−∞_ values (49.09 h for AMXT and 22.65 h for FFC) and reduced Cl/F for both drugs.

The extended T_max_ of AMX relative to its t_1/2_ suggests sustained absorption, likely due to slow release from the injection site depot. These findings indicate that the AMXT–FFC combination enhances systemic exposure and prolongs the duration above MIC for both antibiotics, suggesting its potential as a long-acting injectable formulation for bacterial infections in olive flounder.

### Dose determination and PK/PD analyses

To evaluate the suitability of a 20 mg/kg combination dose (10 mg/kg each of AMXT and FFC) for efficacy testing, PK parameters were integrated with the MIC (0.5 µg/mL) to evaluate antibacterial exposure and dose adequacy following intramuscular administration in olive flounder. Table 2 shows that AMX exhibits rapid absorption and sustained plasma concentrations above the MIC throughout the 48-h observation period. The percentage of time above MIC (%T > MIC) reached 100%, approximately double the standard β-lactam efficacy target (≥ 40%–50%), indicating sustained time-dependent activity. The C_max_/MIC and AUC/MIC ratios were 68.24 and 2,861.80, representing 6.8-fold and 22.9-fold exceedances over the established efficacy thresholds, respectively. These findings indicate potent bactericidal exposure and extended therapeutic coverage throughout the dosing interval.

**Table 2:** PK-PD indices and target attainment following single-agent and combined intramuscular administration of AMXT and FFC in olive flounder

Parameter	AMXT alone (MIC = 4 µg/mL)	FFC alone (MIC = 1 µg/mL)	AMXT combination (MIC = 0.5 µg/mL)	FFC combination (MIC = 0.5 µg/mL)	PK-PD target^a^	Target attainment^b^
C_max_ (µg/mL)	12.73	15.60	34.12	31.59	-	-
%T > MIC (%)	76.30	-	100.00	-	≥ 40–50 (β-lactam)	200% above target
C_max_/MIC	3.18	15.60	68.24	63.18	≥ 8–10 (conc-dep.)	AMXT 6.8×; FFC 6.3× threshold
AUC_0−∞_/MIC (h)	48.46	198.60	2,861.78	1,610.82	≥ 125 (amphenicol)	AMXT 22.9×; FFC 12.9× threshold
PK-PD driver	Time-dependent	Concentration-dependent	Time-dependent	Concentration-dependent	-	-

PK, pharmacokinetics; PD, pharmacodynamics; AMXT, amoxicillin trihydrate; FFC, florfenicol; MIC, minimum inhibitory concentration; PK-PDC_max_, maximum plasma concentration; %T > MIC, percentage of time that drug concentration exceeds minimum inhibitory concentration; AUC_0–∞_, area under the concentration–time curve from time zero to infinity; AUC, area under the concentration–time curve; CLSI, Clinical & Laboratory Standards Institute; EMA, European Medicines Agency.

^a^PK-PD targets based on class-specific efficacy thresholds: β-lactams (%T > MIC ≥ 40%–50%) and amphenicols (AUC/MIC ≥ 125), as defined in CLSI VET03-A and EMA CVMP/VICH GL52 guidelines.

^b^Target attainment indicates the fold-exceedance of PK-PD thresholds associated with bactericidal activity (MIC-adjusted).

For FFC, a concentration-dependent antimicrobial, the combination treatment similarly increased systemic exposure. The C_max_/MIC ratio was 63.18, 6.3-fold higher than the target (≥ 8–10), while the AUC/MIC ratio was 1,610, exceeding the reference value of 125 by 12.9-fold. These PK-PD indices confirmed that FFC achieved optimal PD exposure to maintain bactericidal concentrations for ≥ 48 h. Overall, these findings from the PK-PD integration indicate that a single intramuscular AMXT–FFC dose (10 + 10 mg/kg) maintains plasma concentrations above the MIC throughout the sampling period.

### *In vivo* therapeutic efficacy

#### Lethal dose determination (challenge model qualification)

**Fig. 3:**
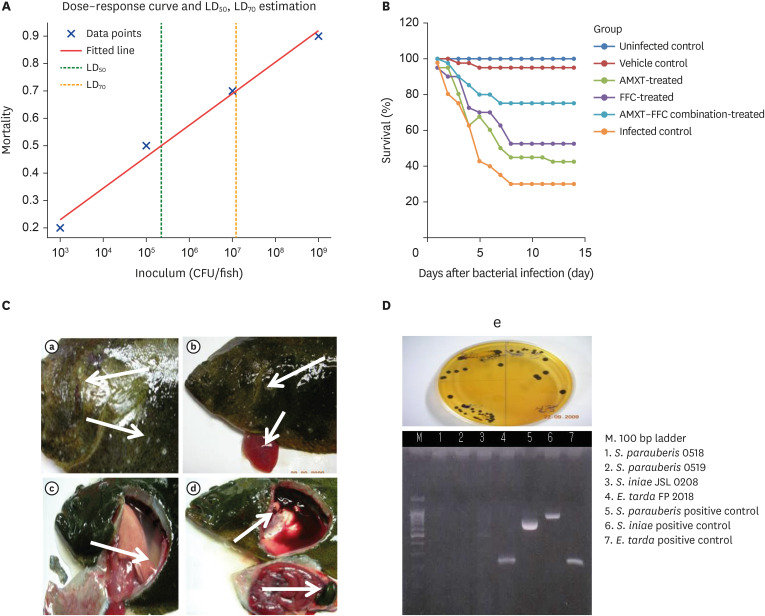
*In vivo* therapeutic efficacy of AMXT, FFC, and their combination in olive flounder experimentally infected with *Edwardsiella tarda* FP2018. (A) Dose–response mortality curve used to estimate LD_50_ (4.24 × 10^5^ CFU/fish) and LD_70_ (3.7 × 10^6^ CFU/fish). (B) Kaplan–Meier survival analysis over 14 days post-challenge and post-treatment (IM, 48 h post-infection). The AMXT–FFC group showed the highest survival (75%; RPS = 65.5) compared to infected controls (27.5%) (log-rank test, *p* < 0.05). (C) Representative gross lesions (white arrows) in infected fish, including fin base hemorrhage, anal bleeding, splenomegaly, hepatic congestion, and renal hemorrhage. (D) Pathogen confirmation using culture and PCR: typical *E. tarda* colonies on TSA and species-specific PCR targeting gyrB; absence of amplification for *Streptococcus iniae* and *Streptococcus parauberis*. M, 100-bp DNA ladder; lanes indicate isolates from examined tissues. AMXT, amoxicillin trihydrate; FFC, florfenicol; LD_50_, median lethal dose; CFU, colony-forming units; LD_70_, dose causing 70% mortality; IM, intramuscular; RPS, relative percent survival; PCR, polymerase chain reaction; TSA, tryptic soy agar; gyrB, DNA gyrase subunit B gene.

To determine an appropriate challenge dose for evaluating therapeutic efficacy, olive flounder were intraperitoneally inoculated with graded doses of *E. tarda* FP2018 (10^3^–10^9^ CFU/fish), and cumulative mortality was monitored for 14 days (Fig. 3A). Mortality increased with bacterial dose, with cumulative deaths of 20%, 50%, 70%, and 90% observed at 10^3^, 10^5^, 10^7^, and 10^9^ CFU/fish, respectively, while no mortality occurred in saline-injected controls. Nonlinear sigmoidal regression of the dose–response data revealed a strong positive correlation between inoculum size and mortality, yielding an LD_50_ of 4.24 × 10^5^ CFU/fish and an LD_70_ of 3.7 × 10^6^ CFU/fish (R^2^ > 0.98). The regression model closely fit the observed mortality data, confirming the reproducibility of the challenge model.

Consequently, the LD_70_ inoculum was selected as the standard challenge dose for subsequent efficacy testing, ensuring consistent systemic infection and measurable treatment effects.

#### Therapeutic efficacy in the E. tarda-infected flounder

Following challenge with the LD_70_ inoculum, the AMXT–FFC combination group (intramuscular administration of 20 mg/kg; 10 mg/kg each of AMXT and FFC administered 48 h postinfection) achieved the highest survival rate over the 14-day observation period (Fig. 3B). Kaplan–Meier survival analysis revealed 75.0% survival in the combination-treated group compared to 27.5% in the infected untreated control group (log-rank *p* < 0.05), corresponding to an RPS of 65.5%. AMXT- or FFC-treated groups showed partial protection, with RPS values of 32.1% and 42.9%, respectively. The combination therapy exceeded the 60% efficacy threshold commonly used to indicate significant therapeutic protection in antimicrobial or vaccine studies. This improved outcome suggests that the combination provides more durable bactericidal control and supports host recovery, with no additional mortalities observed after day 8 posttreatment. Fig. 3B illustrates the sustained protective effect of combination therapy relative to the incomplete responses observed with monotherapy.

Postmortem examinations and bacterial re-isolation results closely matched survival outcomes (Fig. 3C and D). In infected untreated controls, *E. tard*a was recovered at high frequencies from major organs, including the kidney (92.5%), brain (70%), eye (72.5%), and liver (100%), indicating systemic dissemination. Conversely, AMXT–FFC treatment reduced bacterial recovery rates to 25% (kidney), 15% (brain), 17.5% (eye), and 20% (liver). PCR analysis of characteristic black-centered colonies on selective agar confirmed the isolates as *E. tarda* FP2018 (D). Most tissues from treated fish were culture-negative and classified as “protected sites,” consistent with gross pathological improvements, including resolution of hemorrhagic skin lesions, reduced visceral congestion, and normalization of hepatic and renal coloration (Fig. 3C). These findings indicate that intramuscular administration of the AMXT–FFC combination suppresses bacterial colonization and systemic dissemination, thereby promoting tissue recovery and enhanced survival.

## DISCUSSION

Judicious antimicrobial use in aquaculture remains essential to preserve drug efficacy and limit the development of antimicrobial resistance [27,28,29,30,31]. Combination therapy with established antibiotics has been proposed as an antimicrobial stewardship strategy to enhance therapeutic outcomes while minimizing selective pressure [10,32]. Among available administration routes, intramuscular injection offers the most precise dosing and avoids variability in drug intake associated with medicated feed. Within this framework, this study shows that a fixed-dose AMXT–FFC formulation exhibits *in vitro* synergy, alters key PK parameters following intramuscular administration, achieves prespecified PK-PD targets under the experimental conditions, and improves therapeutic efficacy against *E. tarda* in olive flounder.

This study focuses on the well-characterized *E. tarda* FP2018 strain to standardize the PK-PD framework within a consistent infection model. To extend clinical applicability, future research will establish epidemiological cut-off values from diverse field isolates and utilize Monte Carlo simulations to optimize dosing regimens across varying antimicrobial susceptibilities. Time–kill assays confirmed that the AMXT–FFC combination exhibited synergistic bactericidal activity against *E. tarda* at clinically relevant concentrations (1×–2× MIC) (Fig. 1). The ≥ 3 log_10_ CFU/mL reduction observed within 12–24 h reflects the complementary mechanisms of action, while AMX inhibited cell-wall synthesis and FFC suppressed protein synthesis. While > 5 log_10_ CFU/mL reductions were observed at 4× MIC, this may reflect concentration-dependent killing rather than enhanced synergy. Comparable patterns are reported for β-lactam–phenicol combinations, with synergy most pronounced near MIC levels. Static MIC or fractional inhibitory concentration assays alone cannot fully capture the dynamic bactericidal kinetics driven by fluctuating drug concentrations *in vivo* [5,33]. Conversely, time–kill kinetics provide quantitative data on antibacterial activity across concentration gradients, establishing a mechanistic link to PK-PD modeling. The observed synergy is consistent with established mechanistic complementarity, whereby simultaneous inhibition of cell wall synthesis and ribosomal function disrupts parallel bacterial survival pathways [33,34]. Furthermore, the sustained antibacterial effect suggests minimal β-lactamase activity in the FP2018 strain or potential FFC-mediated suppression of β-lactamase expression. These findings suggest the integration of PK-PD analyses and clarify the observed *in vivo* therapeutic outcomes.

The terminal phase observed in this study was characterized by prolonged systemic persistence under the experimental conditions. Formulation-dependent physicochemical properties, including aqueous solubility and dissolution kinetics, influence intramuscular absorption behavior in teleost species [35,36,37]. However, without intravenous reference data or independent estimates of absorption rate constants, the relative contributions of absorption and elimination processes to the terminal decline cannot be resolved. Accordingly, the extended terminal phase reflects apparent prolonged residence time rather than confirmed absorption-limited kinetics. Future studies incorporating IV administration and compartmental modeling are needed to fully elucidate the disposition characteristics of this formulation. Coadministration of AMXT and FFC substantially modified the PK disposition of both agents relative to monotherapy, significantly increasing C_max_ and prolonging t_1/2_ for AMXT, increasing C_max_ for FFC, and extending T_max_ with changes in Cl/F and Vz/F (Table 2; Fig. 2). These characteristics explain the 100% time above MIC (T > MIC) for AMXT observed in this study, an outcome unlikely to occur with standard soluble formulations.

Comparative PK studies in teleost species demonstrate that formulation, administration route, species differences, and ambient temperature significantly affect AMX disposition. Lower drug exposure in tilapia and other warm-water species likely reflects increased temperature-dependent clearance, oral dosing, and the use of sodium salt formulations [23]. The FFC PK profile observed in this study is consistent with those in previous studies in flounder and cod, demonstrating moderate persistence and minimal formation of FFC-amine metabolites [25,26,27,28].

Co-administration modified the PK disposition of both agents, altering peak exposure and apparent kinetics while maintaining plasma concentrations above the synergistic MIC for > 48 h (Fig. 2). This profile reflects the PK-PD characteristics of each agent: time-dependent activity for AMXT and concentration-dependent bacterial activity for FFC [21,28,38].

PK-PD analyses confirmed that the fixed intramuscular dose of 10 + 10 mg/kg provided adequate PD exposure for both agents. AMXT achieved 100% T > MIC at the combination MIC of 0.5 µg/mL, substantially exceeding the classical β-lactam target of 40%–50%. Similarly, FFC exposures markedly exceeded recommended thresholds (AUC/MIC ≥ 125; C_max_/MIC ≥ 10) (Table 2) [38]. These complementary PD mechanisms—continuous inhibition via AMXT and high-intensity exposure via FFC—potentially underlie the observed *in vivo* antibacterial efficacy. From a clinical and practical perspective, the depot-like PK profile achieved with a single intramuscular injection offers clear advantages by maintaining sustained plasma concentrations above the MIC for both agents. This approach effectively overcomes the limitations associated with oral therapy, such as variable feed intake, and ensures prespecified PK-PD targets, thereby providing prolonged, synergistic exposure that enhances survival while minimizing selective pressure [21,24,28]. These findings suggest that the selected dosing regimen achieves the predefined PK-PD targets under controlled experimental conditions. However, variability in field conditions and pathogen susceptibility distributions may influence target attainment in practice. Therefore, further evaluation is warranted under commercial aquaculture conditions to confirm dosing robustness across diverse environmental and epidemiological settings.

Nonetheless, target attainment depends on MIC distributions, water temperature, and species-specific clearance rates. Elevated MIC values or higher temperatures may reduce %T > MIC and AUC/MIC; therefore, ongoing monitoring of local susceptibility patterns and temperature-based dose adjustment is warranted. The AMXT–FFC combination produced substantially improved therapeutic outcomes compared to monotherapy, achieving 75% survival vs. 27.5% in the untreated control group (Fig. 3A). The limited protection achieved with AMXT and FFC monotherapy (32.1% and 42.9%, respectively) is consistent with those of previous challenge studies with single-agent regimens failing to provide complete protection despite *in vitro* susceptibility. In contrast, the combination maintained PK-PD indices above established efficacy thresholds, thereby eliminating exposure gaps that compromise monotherapy effectiveness. Organ-level analyses revealed substantially reduced bacterial recovery in the kidney, brain, eye, and liver (Fig. 3B), correlating with improved survival and reduced lesion severity [5,17,32]. Consistent findings across microbiological, pathological, and survival endpoints indicate that the combination effectively suppresses systemic bacterial dissemination. The LD_70_ challenge model enhanced discrimination among treatment groups and may have contributed to the clear separation of survival outcomes [32]. Under high disease pressure, the combination outperformed both monotherapies, exceeding typical efficacy reported in warm-water teleosts, with β-lactam and phenicol monotherapies often resulting in inconsistent outcomes. Differences between these findings and those of previous studies can be attributed to the following factors: (i) higher FFC AUC/MIC ratios at lower temperatures, (ii) suboptimal %T > MIC for β-lactams in many species, and (iii) variable oral absorption in feed-based studies. The intramuscular administration-maintained plasma concentrations above the MIC for both agents under the present experimental conditions.

Collectively, these findings indicate that a single intramuscular dose of AMXT–FFC provides prolonged antibacterial exposure, resulting in significantly enhanced survival and reduced bacterial colonization of target organs. The alignment of time-dependent β-lactam activity with concentration-dependent FFC effects represents a pharmacologically coherent, stewardship-aligned therapeutic approach for managing edwardsiellosis. A key limitation is the use of group-housed tanks and the limited number of replicate tanks per treatment. Although environmental conditions were standardized, residual tank-level effects cannot be excluded. Future studies incorporating fully replicated, tank-level randomized designs would enable mixed-effects modeling to better quantify between-tank variability. Nonetheless, future studies should prioritize the following: (i) development of population PK models incorporating covariates, including temperature, body mass, and stress; (ii) intravenous studies to better define bioavailability and disposition parameters; (iii) residue depletion studies to establish withdrawal periods; and (iv) regional MIC distribution analyses to support dosing robustness. These efforts will facilitate optimized clinical application and broaden the use of this combination therapy across diverse aquaculture species.

## SUPPLEMENTARY MATERIALS

Supplementary Table 1 Water quality parameters measured during the adaptation period (0–6 days) and the test period (7–28 days) in the olive flounder experimental system

Supplementary Table 2 MICs of AMX, FFC, and their combinations at different ratios

Supplementary Table 3 Statistical comparison of AUC_last_ between single and combined administration groups using Bailer–Satterthwaite method

Supplementary Fig. 1 Method validation data and representative chromatograms. (A) Precision data tables and calibration curves demonstrating linearity (R^2^ = 0.9998) for amoxicillin (0.05–10 µg/mL) and florfenicol (0.1–50 µg/mL). (B) Representative HPLC chromatograms of amoxicillin detected at 229 nm. (C) Representative HPLC chromatograms of florfenicol detected at 224 nm. For both chromatographic panels, traces represent blank plasma (top), spiked plasma (middle), and plasma samples (bottom). Arrows indicate the retention peaks of the target analytes.

## References

[1] Hamidoghli A, Won S, Lee S, Lee S, Farris NW, Bai SC (2020). Nutrition and feeding of olive flounder *Paralichthys olivaceus*: a review. Rev Fish Sci Aquacult.

[2] Ranjan A, Sahu NA, Gupta S, Aklakur M (2017). Prospects of medicated feed in aquaculture. Nutr Food Sci Int J.

[3] Hegde A, Kabra S, Basawa RM, Khile DA, Abbu RUF, Thomas NA (2023). Bacterial diseases in marine fish species: current trends and future prospects in disease management. World J Microbiol Biotechnol.

[4] Baeck GW, Kim JH, Gomez DK, Park SC (2006). Isolation and characterization of *Streptococcus* sp. from diseased flounder (*Paralichthys olivaceus*) in Jeju Island. J Vet Sci.

[5] Janda JM, Duman M (2024). Expanding the spectrum of diseases and disease associations caused by *Edwardsiella tarda* and related species. Microorganisms.

[6] Park SB, Kwon K, Cha IS, Jang HB, Nho SW, Fagutao FF (2014). Development of a multiplex PCR assay to detect *Edwardsiella tarda*, *Streptococcus parauberis*, and *Streptococcus iniae* in olive flounder (*Paralichthys olivaceus*). J Vet Sci.

[7] Bouquin S, Thomas R, Jamin M, Baron S, Hanne-Poujade S, Chauvin C (2021). A baseline survey of antimicrobial use and health issues in the freshwater salmonid industry in France. Aquacult Rep.

[8] Manyi-Loh C, Mamphweli S, Meyer E, Okoh A (2018). Antibiotic use in agriculture and its consequential resistance in environmental sources: potential public health implications. Molecules.

[9] Defoirdt T, Sorgeloos P, Bossier P (2011). Alternatives to antibiotics for the control of bacterial disease in aquaculture. Curr Opin Microbiol.

[10] Sullivan GJ, Delgado NN, Maharjan R, Cain AK (2020). How antibiotics work together: molecular mechanisms behind combination therapy. Curr Opin Microbiol.

[11] Angst DC, Tepekule B, Sun L, Bogos B, Bonhoeffer S (2021). Comparing treatment strategies to reduce antibiotic resistance in an *in vitro* epidemiological setting. Proc Natl Acad Sci U S A.

[12] Stratev D, Odeyemi OA (2016). Antimicrobial resistance of *Aeromonas hydrophila* isolated from different food sources: a mini-review. J Infect Public Health.

[13] Watts JEM, Schreier HJ, Lanska L, Hale MS (2017). The rising tide of antimicrobial resistance in aquaculture: sources, sinks and solutions. Mar Drugs.

[14] Wang X, Sun B (2023). Metabolic proteins with crucial roles in *Edwardsiella tarda* antioxidative adaptation and intracellular proliferation. mSystems.

[15] Park SB, Aoki T, Jung TS (2012). Pathogenesis of and strategies for preventing *Edwardsiella tarda* infection in fish. Vet Res.

[16] Turnidge JD (1998). The pharmacodynamics of β-lactams. Clin Infect Dis.

[17] Abayneh T, Colquhoun DJ, Sørum H (2013). Edwardsiella piscicida sp. nov., a novel species pathogenic to fish. J Appl Microbiol.

[18] Li M, Wu M, Sun Y, Sun L (2022). Edwardsiella tarda TraT is an anti-complement factor and a cellular infection promoter. Commun Biol.

[19] Sun B, Zhang M (2016). Analysis of the antibacterial effect of an *Edwardsiella tarda* LuxS inhibitor. Springerplus.

[20] Prescott JF, Giguère S, Prescott JF, Dowling PM (2013). Antimicrobial Therapy in Veterinary Medicine.

[21] Park JY, Awji EG, Suh JW, Park SC (2016). Pharmacokinetics, pharmacokinetic-pharmacodynamic relationship, and withdrawal period of amoxicillin sodium in olive flounder (*Paralichthys olivaceus*). Xenobiotica.

[22] Phu TM, Em NT, Thinh NQ, Phuong NT, Dalgaard A, Scippo ML (2019). Pharmacokinetics and muscle residue depletion of amoxicillin in cage cultured hybrid red tilapia (*Oreochromis mossambicus* × *Oreochromis niloticus*). Aquaculture.

[23] Rairat T, Lu YP, Ho WC, Ke HJ, Chou CC (2024). Pharmacokinetics, optimal dosages and withdrawal time of amoxicillin in Nile tilapia (*Oreochromis niloticus*) reared at 25 and 30 °C. Vet Q.

[24] Seo JS, Jeon EJ, Jung SH, Park MA, Kim NY (2015). Pharmacokinetics of amoxicillin trihydrate in cultured olive flounder (*Paralichthys olivaceus*). J Vet Pharmacol Ther.

[25] Horsberg TE, Hoff KA, Nordmo R (1996). Pharmacokinetics of florfenicol and its metabolite florfenicol amine in Atlantic salmon. J Aquat Anim Health.

[26] Park BK, Lim JH, Kim MS, Yun HI (2006). Pharmacokinetics of florfenicol and its metabolite, florfenicol amine, in the Korean catfish (*Silurus asotus*). J Vet Pharmacol Ther.

[27] Samuelsen OB, Bergh O, Ervik A (2003). Pharmacokinetics of florfenicol in cod *Gadus morhua* and *in vitro* antibacterial activity against *Vibrio anguillarum*. Dis Aquat Organ.

[28] Sidhu P, Rassouli A, Illambas J, Potter T, Pelligand L, Rycroft A (2014). Pharmacokinetic-pharmacodynamic integration and modelling of florfenicol in calves. J Vet Pharmacol Ther.

[29] Clinical and Laboratory Standards Institute (CLSI) (2020). CLSI Guideline VET03. Methods for Antimicrobial Broth Dilution and Disk Diffusion Susceptibility Testing of Bacteria Isolated From Aquatic Animals.

[30] European Medicines Agency (EMA) Guideline on demonstration of target animal safety and efficacy of veterinary medicinal products intended for use in farmed finfish. EMA/CVMP/EWP/459868/2008.

[31] Luu QH, Nguyen TBT, Nguyen TLA, Do TTT, Dao THT, Padungtod P (2021). Antibiotics use in fish and shrimp farms in Vietnam. Aquacult Rep.

[32] Lee SJ, Park SC (2015). Amoxicillin–florfenicol combination reduces mortality in olive flounder (*Paralichthys olivaceus*) experimentally infected by *Streptococcus iniae*. Aquacult Res.

[33] Lee EM, Choi M, Lee SJ, Park SC (2010). Pharmacodynamics of florfenicol alone and in combination with amoxicillin or cefuroxime against pathogenic bacteria of fish origin. Korean J Vet Res.

[34] Kim S, Woo JH, Jun SH, Moon DC, Lim SK, Lee JC (2020). Synergy between florfenicol and aminoglycosides against multidrug-resistant *Escherichia coli* isolates from livestock. Antibiotics (Basel).

[35] Yáñez JA, Remsberg CM, Sayre CL, Forrest ML, Davies NM (2011). Flip-flop pharmacokinetics-delivering a reversal of disposition: challenges and opportunities during drug development. Ther Deliv.

[36] Ladaidi A, Hallez L, Pochard I, Rouge N, Hihn JY (2025). The effect of ultrasound on the crystallization-precipitation process of transforming sodium amoxicillin into amoxicillin trihydrate. Ultrason Sonochem.

[37] Fernandez C, Modamio P, Mestorino N, Errecalde JO, Mariño EL (2007). Pharmacokinetics of sodium and trihydrate amoxicillin in sheep after intravenous and intramuscular administration. J Vet Pharmacol Ther.

[38] McKellar QA, Sanchez Bruni SF, Jones DG (2004). Pharmacokinetic/pharmacodynamic relationships of antimicrobial drugs used in veterinary medicine. J Vet Pharmacol Ther.

